# Hospital and Institutional News

**Published:** 1919-09-20

**Authors:** 


### HOSPITAL AND INSTITUTIONAL NEWS.

### PREVENTION OF TUBERCULOSIS.

The National Association for the Prevention of Tuberculosis has issued a provisional programme of the seventh annual Conference to be held at the Central Hall, Westminster, S.W., on October 16, 17, and 18. The completion of tuberculosis schemes throughout the country will be considered in relation to the Ministry of Health, Local Authorities and Insurance Committees, Pensions Boards, General Practitioners, Red Cross and other voluntary activities, and Training of Doctors and Nurses. On October 16, at the morning session, Dr. Addison will give the opening address, and this, together with the afternoon session, will be devoted to the first four of the above-mentioned subjects. Among others, Sir R. W. Philip, Dr. Hermann Biggs of New York, and Lieut.-Col. Nathan Raw will speak. On the next day, under the chairmanship of Sir Arthur Stanley in the morning, and Sir R. W. Philip in the afternoon, the last two subjects will be considered, and both Sir Wm. Osler and Col. Sir G. Sims Woodhead are expected to give addresses. On the third day Tuberculosis Work for the Red Cross and other workers will form the subject at a morning session commencing at 10.30 a.m. Together with other social functions in process of arrangement, there are announced a Conversazione on the evening of October 16 and an At Home—Lady Glenconner—October 17 (evening).

### DISABLED MEN.

As from September 3 the revised scale of disablement pensions for the higher ranks, affecting soldiers and airmen, came into force, an increase of 17 per cent. to 19 per cent., varying with the rank, is provided on the previous rate for total disablement, in addition to allowances for wife and children. Various alterations and adjustments have also been made for widows of men in all Services. The new rates will continue for at least three years, and will, therefore, be subject to readjustment according to the cost of living, but will not under any circumstances be lowered more than 20 per cent. or under the previous rates. The Ministry of Labour and the Ministry of Pensions announce that War Pensions Committees are authorised, where recommended by the medical referee, to continue to pay allowances to disabled men, in training under the Ministry of Labour, over and above the training allowances which they receive from the Ministry of Labour for "constant attendance" and special diet. Also, the divisional directors of the Ministry of Labour are not responsible for granting or payment of these allowances, which come under the heading of treatment, and any disabled men who desire their continuation or authorisation should apply to their War Pensions Committees.

### LOCAL HEALTH STAFFS.

The local authorities have been circularised by the Ministry of Health, asking for particulars of the staffs engaged in medical, sanitary and housing work. Included in the return are to be all medical men and women, all sanitary inspectors, the staff of the surveyor's department, housing inspectors, and any other officers appointed at a yearly salary and whose duties are concerned with public health. It is also asked that indication be made whether there is a scale of war bonus which applies to all employees, and, if so, to state the full details.

### PUBLIC HEALTH IN IRELAND.

The chief secretary has nominated the following to be members of the Irish Public Health Council under the Ministry of Health Act 1919:—Dr. E. Coey Bigger to be chairman of the Council, together with Sir John W. Moore, President of the Royal Academy of Medicine of Ireland; Dr. Robert J. Rowlette; Miss Alice Barry, M.R.C.P., medical superintendent of the Child Welfare Work Branch of the Women's National Health Association; Lady Kenmare, chairman of the Advisory Council for Ireland of the Queen Victoria Jubilee Institute; Mrs. J. McMordie, member of the Tuberculosis Committee of the Belfast Corporation; Sir James M. Gallagher, ex-Lord Mayor of Dublin; Mr. J. Ewing Johnston, M.B.E., M.R.C.V.S., President of the North of Ireland Veterinary Medical Association; the Rev. P. Kerlin, C.C., Bellarena, Derry; and Mr. John Drennan, representing the Approved Insurance Societies. The following are *ex-officio* members of the Council:—The Vice-President and the two other Commissioners of the L.G.B. for Ireland, the Chairman and two other Irish Insurance Commissioners, and the Registrar-General of Births, etc. Major George A. Harris, D.S.O., has been appointed Secretary of the Council.

### HEALTH MINISTRY IN U.S.A.

Before the United States Congress there are now two Bills for the establishment of a National Health Department. We understand that the Bills are, in their main outline, similar, providing for a Secretary of State, who would be a member of the Cabinet, and a Commissioner of Health. The department would include bureaux of sanitary research, child welfare, vital statistics, food and drugs questions, quarantine and Government hospitals, &c.; an advisory board of seven expert consultants; and a committee on local health matters. As in this country, sundry at present out-lying departments would be transferred to the national health organisation.

### INFLUENZA AND PROPHYLAXIS.

In support of the use of prophylactic inoculations of mixed vaccines against influenza we read some interesting notes from the pen of Surgeon-Lieut. F. T. Grey, R.N., O.C. Samoa Relief Expedition in 1918, and published in the *Lancet* of September 13. The following evidence in support of systematic prophylaxis is, as the author states, impressive to say the least of it. The entire ship's company of the man-o'-war which took his expedition to the islands were inoculated. Shore communication was restricted as far as possible, but, in spite of the ideal being unattainable, not one case of influenza developed. Every member of the expedition was inoculated at least four times in three months. Not a single case developed, although the risk of infection was no small one, in that Samoa alone one-fifth of the entire population was wiped out by the scourge. To test the efficacy of the vaccine, opportunity was provided by forty natives happening to be sent from a clean to an infected area during the progress of the epidemic. These were isolated, inoculated, and not released until judged to be in a positive phase. Not one contracted the disease. Other examples are given in the instance of an incoming passenger steamer and the naval depôt at Williamstown, and in every instance the favourable evidence is remarkably strong, although further details of the local bacteriology would be advisable before applying the lesson directly to home conditions.

### THE NOTE ON VENEREAL DISEASE.

Recently the Ministry of Health issued as a White Paper a Report of the Inter-Departmental Committee appointed last January to consider the question of measures to be taken against the dangers of dissemination of tropical and other diseases in this country. At the time space did not allow of comment in this journal, but there are certain points raised which will draw considerable attention even if they do not relight the flame of controversies which have until very recently raged. The present memorandum is concerned mainly with the advisability or otherwise of making officially available among the civil community certain methods of prophylaxis against venereal diseases —the "packet" system so-called—which have been stated as successful among the combatants during the war. The general conclusion, however, states that "they are not satisfied that there has been sufficient evidence put before them of the beneficial results gained by the distribution of prophylactic packets in various Forces to prove the value of the system, or to justify them in recommending its official encouragement among the civil population." This conclusion, it may be noted, was issued by the Chairman, Major Astor, and not signed by the members. In the main, the memorandum is devoted to a review, well supported by figures, of the good effect produced by recreation grounds, libraries and a number of innocent amusements for the men. The benefit derived from education and straight talking by medical officers on the subjects of common-sense, culture, health and self-restraint is proved over and over again by adequate evidence taken from the British, Colonial and United States Army Corps, but the whole line of argument impresses us as being taken largely from the moral standpoint. Also the lay press are considerably divided in their manner of receiving this decision. On the one hand, we see it stated that the Committee, having covered the whole ground of the important questions involved, we must therefore regard the conclusions at which they have arrived upon the subject of prophylaxis as *settling* a controversy in the course of which, at times, acrimony was a distinguishing feature. On the other, a famous contemporary describes the whole as a document which is halting with respect to strictly scientific matter and unrestrained in its confusion between moral and medical precepts.

### NEED FOR TECHNICAL OPINION.

We have no intention of reopening in these pages a discussion of this somewhat painful subject, but would nevertheless point out that, in agreement with the latter opinion, we feel that this memorandum, definite though its ultimate conclusion may be, leads up to it in a manner more than a little confusing. One may find a number of points of weakness, and they fall invariably on the technical side of the subject. Full distinction even is not drawn between the two common venereal diseases. Indirectly doubt is thrown upon the efficiency of our accepted prophylactic means, but without, as far as one can detect, any real evidence being brought against them. There is distinct confusion between the requirements of prophylaxis and treatment, and one cannot readily appreciate the supposed dangers which lie in the unskilled use of the packet. As the *Times* remarks, these measures are "not absolute, but neither is it an absolute protection against tetanus when we wash our hands after gardening, or hold a cut finger under the tap; . . . men are often careless and forget to use the prophylactic; or drunk, and therefore reckless. Precisely similar causes have kept men from following many simple rules of life, but they are not usually accepted as evidence against these rules." Throughout the note in all arguments put forward, there is found the idea that the general use of prophylactic means must remove an otherwise occurring and beneficial restraint. But actually, if *prevention* of the spread of these diseases is kept in view as the object of paramount importance to the country, then it appears that these arguments lead rather away from than towards it. From one aspect of the subject a definite conclusion has been drawn, and from that aspect it may be very largely correct, but, before final acceptance, we feel that the result should be weighed against carefully thought-out advice from a Committee technical both in its knowledge and its outlook, and fuller use made of evidence which the medical profession has collected during its varied experiences of the last few years.

### DISTRICT HOSPITALS AND THEIR CENTRE.

We lately recorded the suggestion that the efforts to reduce the shortage of beds at King Edward VII. Hospital, Cardiff, should include the creation of local hospitals to serve the needs of the remoter districts from which the patients now come. There is interest, therefore, in the scheme for a new hospital for the Mountain Ash district, for which the present cottage hospital is too small to provide, with the result that local cases have often had to be sent to Cardiff. The sum of £1,600 has been subscribed, including the result of a levy of sixpence per head from workers in the local pits and collieries. A site near the Grove, Mountain Ash, is the subject of negotiation, and it is hoped eventually to provide forty beds. The progress made does honour to the temporary Chairman, Mr. John Jarvis, to Mr. W. T. Bowen, the Secretary, and to Mr. George Hall, the Treasurer. Great central institutions can never do all the work of a large, populous district. The local hospitals are necessary to schemes of which the great hospitals are the centres. The advantage of the voluntary system is precisely its adaptability to circumstance and local feeling.

### MEMORIAL TO COLONEL BRUCE-VAUGHAN.

THAT the friends of King Edward VII. Hospital, Cardiff, would wish to commemorate the late Colonel E. M. Bruce-Vaughan was certain, and that their wish should take the form of completing the great work which he initiated was, we are glad to think, inevitable. Under General Lee the board has now decided to appeal for £60,000 by way of memorial to Colonel Bruce-Vaughan, and this sum has been chosen because the sum needed to pay the overdraft on the present extensions, and the sum needed to complete them amount together to that figure—a very modest sum for Cardiff, by the way. The late Colonel's appeal realised £38,500, and if he had lived, his influence and energy would certainly have secured the whole sum. One of the rewards of his success was the present overdraft of £25,000, for the bank would not have yielded to his plea that the work must be carried out first and paid for afterwards, if the spirit behind the plea did not guarantee a power to do both. With the new medical school, and the recent extensions, all Wales is interested in the success of the work which Colonel Bruce-Vaughan fostered so zealously. It is in accordance with the impetus that he bequeathed that an effort should be made to complete it. We congratulate Colonel Lee and his colleagues on their policy. It is in the fine tradition of the work already done. The surprise rather is that none of the prosperous men of Cardiff, who have been enriched by the war, has not sent a cheque for the whole sum already. There is no difficulty about it. The money is there. The honour is waiting.

### SIR HAROLD PINK AND PORTSMOUTH HOSPITAL

THE difficulties of the Portsmouth Hospital are an old story, and the next step, so to speak, is indicated by Sir Harold Pink's endeavour to introduce a larger share of popular control. He wishes to reconstruct the board, and proposes that it should consist of forty-two members, twelve to be elected from subscribers by themselves, and twelve by subscribers from candidates submitted by the Naval Barracks (2), R.M.A. (1), R.M.L.I. (1), and Trades Council (2), while the remaining six would represent the Dockyard Workmen's Committee. The Corporation, the Guardians, the Gosport Hospital Saturday Fund, and similar bodies would have one representative apiece, and the Committee would also include one Vice-President, the Treasurer, four members of the medical staff, the honorary architect, and the chaplain. Sir Harold Pink also favours workpeople's committees in each district, and since each district committee would submit its own patients, the abuses of the ticket system would be curtailed. All this is promising, and only by the spirit which lies behind such a proposal can the lost support of the public be won. When it has been won there will be little difficulty in paying for the extension, which all desire who know how the shortage of beds has been felt. We have sometimes been reckoned severe critics, but our case merely was that the long failure of the hospital's policy argued a defect in its authors. Such a sign of healthy life as Sir Harold Pink's proposals is therefore reassuring.

### THE MANOR MENTAL HOSPITAL.

NOW that the London County Council institution for the insane is to be known as the Manor Mental Hospital, and no longer the Manor Asylum, all or almost all the County Council institutions of this class receive the new title. This change of name in regard to institutions for mental cases used to annoy the late Dr. C. A. Mercier, and he spent much of his wit in ridiculing it. We defended the change, not only because the new name, for a time at least, will gratify the relations of the patients, but because we supported Dr. Mercier's own plan for the early treatment of mental cases. When the treatment is early enough many cures can be made, and an institution which cures as well as houses patients is entitled to be called a hospital. Since people cannot be argued out of their feeling that mental disease is a disgrace, their feelings should be considered somewhat tenderly. Such consideration may mean a change of name which may have to be repeated, but if we can accompany the change of name by a real change of character there is little to be said against it. In the case of the Manor Hospital, its reopening, since its career as a war hospital was over, is a convenient time to rechristen it, and Dr. Litteljohn, the medical superintendent, is no doubt as eager as the best of his colleagues to promote the early treatment of mental disease.

### RELIGION, THE BODY AND HEALTH.

AMONG the speakers at the now famous series of addresses at St. Martin-in-the-Fields was lately the Rev. Harold Anson, the Warden of the Guild of Health. In speaking of the effect of religion on health, and its relation to medicine (on which an interesting letter from the Guild's then chaplain, Mr. Charles Gardner, appeared in THE HOSPITAL in 1914), Mr. Anson said: "If people are going to find popularity and success in business by mental means, how is it that religion has not been discovered to be the best mental method by which popularity, success, and good health can be secured?" It was, he continued, the success of Christian science to apply religion through mental channels to the body, and the Church could apply it, as it had done in primitive times, again to-day. Those who have studied the matter know that this is more than a vulgar plea for "pelmanising religion." The well observed fact that a patient must have confidence in his doctor, if the latter's treatment is to be fully successful, is a simple instance of the relation of the mind to health and treatment. But we need to know more of that relation. It is not enough to get good results even. The "how" is no less important than the "what." It is for the Guild of Health to undertake the study from its own side, that the medical profession may have less elusive material to consider than has been the case hitherto.

### THE COST OF LUNATICS.

In the last issue of The Hospital the increased cost of lunatics in the care of the London County Council was mentioned. A return has been prepared by Mr. A. P. Stanwell Smith, clerk to the Southwark Board of Guardians, showing the gradual increase in the charge made by the London County Council since 1909. On July 1 in that year, the charge was 9s. 11d. per patient per week, so that, with the proposed charge on October 1 next, of 26s. 10d., there has been an increase in ten years of 16s. 10d. per patient per week. Until September 1, 1912, the charge remained at 9s. 11d., when it went up to 10s. 2½d. and has steadily mounted since April 1914, when it was 11s. 1d., to 16s. 4d. on June 1, 1918. Then came the sudden and large increase of the present year. On April 1, 19s. 10d. was the charge fixed: on July 1 it rose to 23s. 4d.: and on October 1 next it will be 26s. 10d. Southwark has 1,042 patients chargeable in mental hospitals, so that the extra cost of 10s. 6d. a week per patient means that in the next six months £13,000 more will have to be provided out of the rates to meet this increased demand. In Wandsworth £16,500 is required to meet the additional demand, and in Lambeth £16,000.

### A NEW CHANNEL FOR ADVERTISING.

The Salvation Army recently inserted an appeal for funds among the advertisements on a London theatre programme. This is certainly something of a novelty in advertising, but to whom better than those enjoying themselves could an appeal on behalf of the needy be made? "Why not," asks a clerical correspondent, "insert appeals for local hospitals and institutions in parish magazines?" The writer adds this comment, "The church and church people do not know enough of the work of the voluntary hospitals and do not give them sufficient financial support." The parish magazine is something of a joke to non-parishioners, but the clergy ought to know its value, and if it had no value, would it endure?

### THE DECISIVE ADVERTISEMENT.

Signs of healthy activity are evident at the York County Hospital, which has not only more than raised the £3,500 appealed for earlier in the year, but has shown its public spirit by reopening twenty beds in the hut once used for military cases, so that women surgical cases may not have to linger on the waiting-list. The consequence of this step should certainly be, directly, to attract the £2,000 which the new beds will cost to maintain, and indirectly to confirm the confidence which the success of the appeal shows to exist. The best advertisement of any voluntary hospital, and the only finally decisive one, is the value of the work which it performs Now that the York County Hospital has extended its services, we believe that the difficulties of which it used to complain will vanish. Icicles do not melt faster in the sunlight than difficulties and debts in the warmth of vigorous work.

### HOSPITAL STAFFS AND THE SPECIAL POLICE.

The question has arisen as to whether Boards of Guardians should grant permission to their officers engaged in institutional work the privilege of joining the special police reserve. Those wishing to join must be willing to attest for three years' service in the force, and each one must give an undertaking that he will attend the police station to which he is attached, or elsewhere, whenever summoned to do so, and proceed to such place in the Metropolitan area as the Chief Commissioner of Police may decide. The Southwark Board of Guardians has decided not to grant its officers permission to join the police reserve. Whilst fully recognising the important work done by the special police during the war, the guardians were of opinion that, now the emergency was over, it was no longer necessary for public officers to be called away from their work for special police duty, and that their officers should now concentrate on their institutional work.

### PETTY THEFTS AND WARD SISTERS.

With the increase in the cost of commodities there has been a gradual rise in petty thefts from infirmaries and hospitals. These, as a rule, have been confined to linen and apparel, and a recent case in which the Lambeth Board of Guardians prosecuted a night orderly showed how easily goods can be removed. Dr. Lionel Baly, the medical superintendent, in a report to the visiting committee, stated that the matron was compiling a new system of book-keeping, so that the ward sisters would be able to keep a closer watch on their stock. At present it was extremely easy for small articles of clothing to be taken out of the infirmary either by patients, friends, or non-resident officers. It is to be hoped that this will result in a more efficient check being kept on the stock. It is extremely difficult to stop this pilfering, and ward sisters have enough to do without adding to their work the duties of a detective.

### THIS WEEK'S DRUG MARKET.

There is some sign of improvement in the drug market, and business seems to be settling down notwithstanding the continuance in many cases of extreme prices. The item that is attracting most attention at the moment is quinine; with the control removed and in view of shortage of the manufactured article and the increase in the cost of production a considerable increase in price is inevitable. The value of bromides is firmly maintained, but buyers are not disposed to take in large quantities at the moment. Senna leaves are considerably dearer. The value of camphor is firmly maintained with prospects of a further advance. Higher prices are quoted for cod-liver oil. The scarcity of ergot of rye continues and prices have an advancing tendency. Ipecacuanha tends upwards in price. Sugar of milk is slightly cheaper.

